# Psychometric evaluation and cross-cultural adaptation of the Chinese version of the Perception and Understanding of Human Dignity in Nursing Scale: a methodological study

**DOI:** 10.1186/s12912-026-04525-y

**Published:** 2026-03-07

**Authors:** Xuan Qiao, Haixia Zhao, Yiming Lu, Shasha Gao, Huijun Zhang

**Affiliations:** School of Nursing, Jinzhou Medical University, Jinzhou, 121001 China

**Keywords:** Nursing students, Dignity, Perception, Understanding, Psychometric evaluation

## Abstract

**Background:**

Human dignity is a core value in nursing practice. However, there is currently a lack of validated measurement tools in China for assessing the perception and understanding of human dignity in nursing contexts. This study aimed to translate the Perception and Understanding of Human Dignity in Nursing Scale into Chinese and evaluate its psychometric properties among undergraduate and postgraduate nursing students in China.

**Methods:**

This methodological study employed a cross-sectional design. Using convenience sampling, 530 nursing students were recruited from three medical universities in China. The scale was adapted into Chinese following the Brislin back-translation method. Item analysis and content validity evaluation were conducted. The total sample was randomly split into two subsamples for exploratory factor analysis (EFA) and confirmatory factor analysis (CFA) to examine the scale’s latent structure. The scale’s internal consistency reliability, split-half reliability, and test-retest reliability were also assessed.

**Results:**

Item analysis resulted in the retention of 43 items. The scale-level content validity index (S-CVI/Ave), as rated with high consensus by experts, was 1.00. EFA extracted three factors (Understanding dimension, Perception dimension, and Caring dimension), which cumulatively explained 72.455% of the total variance. CFA results indicated acceptable model fit (χ²/df = 2.566, RMSEA = 0.077, SRMR = 0.014, CFI = 0.933, NFI = 0.894), supporting acceptable construct validity. The scale demonstrated a Cronbach’s α coefficient of 0.958, a split-half reliability of 0.836, and a test-retest reliability of 0.966, indicating good reliability within the study sample.

**Conclusion:**

The Chinese version of the Perception and Understanding of Human Dignity in Nursing Scale demonstrated acceptable to good psychometric properties among nursing students in China. It can serve as a preliminary valid tool for assessing nursing students’ perception and understanding of human dignity, providing support for dignity-centered nursing education. Further validation in broader and more diverse clinical nursing populations is warranted in the future.

**Supplementary Information:**

The online version contains supplementary material available at 10.1186/s12912-026-04525-y.

## Background

Respecting and upholding human dignity is a core principle of nursing ethics and an essential characteristic of high-quality care [1]. This consensus stems not only from the ethical requirements of the nursing profession but also from the broad recognition of dignity as a crucial psychosocial determinant of health [2]. However, “dignity,” as a multidimensional and complex sociocultural construct, is profoundly influenced by specific cultural value systems in terms of its interpretation and assessment [3]. This cross-cultural variability poses a fundamental challenge to developing culturally adaptive tools for assessing dignity in nursing. In the field of international nursing research, the measurement of dignity primarily revolves around three types of subjects and contexts. Most of these tools were developed and validated within Western individualistic cultural backgrounds: first, tools assessing patients’ dignity experiences, such as the Patient Dignity Inventory developed by Chochinov et al., which demonstrates good internal consistency in palliative care settings and is mainly used to measure the personal dignity perceptions and related distress of patients with advanced illnesses [4]; second, tools assessing the professional values and ethical competence of nursing staff, such as the Nurses Professional Values Scale, whose Persian revised version exhibits stable factor structures and good reliability and validity, serving as a standardized instrument for assessing professional values among nursing students, applicable in both nursing education and clinical practice contexts, and providing an evaluation basis for optimizing nursing education and enhancing professional practice [5]; and third, tools assessing dignity-related autonomy functions for specific populations or contexts, such as the Maastricht Personal Autonomy Questionnaire (MPAQ). This instrument has undergone cross-cultural adaptation and validation in Spanish and is suitable for assessing the personal autonomy of community-dwelling older adults with chronic multimorbidity, having demonstrated good validity and reliability within this specific population and possessing sound psychometric properties [6]. The construction of these measurement tools is deeply rooted in the Western bioethical paradigm, which emphasizes personal autonomy, the right to self-determination, and privacy [7].

In stark contrast to the aforementioned paradigm, the connotations and maintenance mechanisms of dignity exhibit marked differences within East Asian cultural contexts deeply shaped by collectivism and Confucian ethics. Within such cultures, the conceptualization of dignity places stronger emphasis on an individual’s specific position within social relational networks (e.g., family, community), the fulfillment of corresponding role-based obligations, and the preservation of “face” and the cultivation of harmonious relationships in interpersonal interactions. For instance, the “face-favor” theoretical framework grounded in Confucian relational ethics provides a profound account of the dynamic psychological and social mechanisms that underpin the maintenance of dignity in Chinese social interactions [8, 9]. Meanwhile, in Japanese medical practice, the informed consent process often features a family-centric “negotiated autonomy” decision-making model, rather than an absolute emphasis on individual independent choice [10]. These cultural distinctive features suggest that dignity constructs and their associated measurement tools, derived from Western individualistic philosophical traditions, may fail to accurately and comprehensively capture the complex nuances of dignity perceptions within Chinese nursing contexts due to construct non-equivalence. Nursing education in China faces a prominent methodological bottleneck. Although enhancing nursing humanistic competence has become a clear policy direction [11], and empirical research confirms the positive effect of educational interventions on improving nursing students’ ethical sensitivity [12], there is still a lack of a systematically cross-culturally adapted, Chinese-language standardized tool with rigorous psychometric evidence, specifically designed to assess nursing students’ perception and understanding of the core ethical concept of “human dignity.” Existing Chinese studies predominantly rely on qualitative research methods, such as exploring the authentic experiences of internship nursing students participating in patient care needs surveys through semi-structured interviews [13], or examining the specific learning experiences of nursing students in narrative humanistic education environments [14]. Additionally, some studies employ self-developed questionnaires for measurement, such as the Chinese version of the Dignity Scale in the Perinatal Period developed for specific clinical contexts [15], and the general Public Dignity Perception Scale constructed from a sociological perspective [16]. However, the conclusions of qualitative research inherently have limitations in cross-sample comparison and generalizability. While self-developed questionnaires have undergone preliminary reliability and validity testing, their development was not centered on the nursing student population, lacked systematic cross-cultural adaptation and validation tailored to the Chinese cultural and nursing education context, and their measurement constructs failed to specifically and precisely focus on nursing students’ universal perception and understanding of the core ethical concept of “human dignity.” Consequently, standardized tools specifically designed to measure dignity perception among Chinese nursing students remain scarce. Against this background, the Scale of Perception and Understanding of Human Dignity in Nursing, specifically developed by Yıldırım et al. for nursing education contexts, demonstrates unique value [17]. This scale systematically assesses nursing personnel’s cognition, attitudes, and behavioral tendencies related to dignity across three dimensions: “Perception,” “Understanding,” and “Care.” Its theoretical model aligns with the internalization process of ethical cognition. However, this tool has not yet been introduced into the Chinese context, and its conceptual relevance, reliability, validity, and measurement applicability within the Chinese cultural background remain unknown.

Therefore, this study aims to systematically develop a Chinese version of this scale following internationally recognized cross-cultural adaptation guidelines and comprehensively validate its reliability, validity, and cultural applicability among nursing students in China. The goal is to address the current research gap and provide a scientific and reliable localized measurement tool for evaluating the effectiveness of nursing ethics education and related research in China.

## Methods

### Study design and participants

This study employed a cross-sectional survey design. Data were collected from August to September 2025. A purposive convenience sampling method was adopted. Participants were recruited from three universities located in different geographical regions of China: a comprehensive university in Liaocheng, Shandong Province (East China), which primarily offers junior college nursing education, and two medical universities in Jinzhou, Liaoning Province (Northeast China), which primarily offer undergraduate nursing education. The selection of these specific institutions was based on the following rationale: (1) their nursing programs are accredited by the Ministry of Education, ensuring educational standardization; (2) their geographical distribution (East and Northeast China) and educational levels (junior college and undergraduate) were intended to capture some diversity within the constraints of convenience sampling; (3) existing teaching or research collaborations with the research team ensured feasibility of access and data collection. Furthermore, one of the medical universities offers graduate nursing education, allowing for the potential inclusion of a small number of postgraduate students to preliminarily explore sample heterogeneity.

The study population consisted of nursing students enrolled in the above institutions. Inclusion criteria were: (1) age ≥ 18 years; and (2) voluntary participation with signed informed consent. Exclusion criteria were: foreign students with difficulty understanding the Chinese version of the questionnaire.

Based on standard principles for sample size estimation in reliability and validity studies, the ratio of questionnaire items to sample size should not be less than 1:5 [18]. The questionnaire used in this study contained 43 items, resulting in a minimum required sample size of 215 participants. Considering approximately 20% potential invalid or missing responses and to ensure the stability of factor structure analyses, the final required sample size was set at no fewer than 269 participants. A total of 530 valid responses were collected and included in the study. It is acknowledged that convenience sampling carries a risk of selection bias, which may limit the representativeness and generalizability of the findings.

To avoid potential overfitting in factor analyses and to ensure independence between exploratory and confirmatory factor analyses, the total sample (*n* = 530) was randomly divided into two subsamples, with one group (*n* = 265) used for exploratory factor analysis and the other group (*n* = 265) for confirmatory factor analysis. The number of graduate nursing students was very limited (*n* = 10). Although they were included to enrich the sample background, their representation is severely inadequate. Therefore, no independent subgroup analysis was conducted for this population. All factor analyses were conducted using the total sample, including junior college, undergraduate, and graduate students. This limitation implies that the findings primarily reflect the characteristics of undergraduate and junior college students, and their applicability to the postgraduate population requires further investigation.

## Tools

### General information questionnaire

Through reviewing relevant literature, the researcher independently designed a general information questionnaire. The questionnaire content includes age, gender, educational background, and whether the respondent has taken ethics courses.

### The Scale Perception and Understanding of Human Dignity in Nursing (Chinese version)

The Chinese version was adapted from the original “Scale of Perception and Understanding of Human Dignity in Nursing” developed by Yıldırım et al. [17]. Following authorization from the original author, the adaptation followed the Brislin translation model. The translation process emphasized the consistency of item comprehension within the Chinese context, striving to preserve the original measurement intent (conceptual equivalence) while adapting the wording to the context of nursing education and practice in China. The original scale consists of 43 items across three dimensions: Understanding (Items 1–15), Perception (Items 16–28), and Care (Items 29–43). All items are rated on a 5-point Likert scale ranging from 1 (“strongly disagree”) to 5 (“strongly agree”). It must be explicitly stated that the scale contains no reverse-scored items. All items are scored in the same direction, with higher total and dimension scores indicating a higher level of perception and understanding of human dignity. The original scale demonstrated good internal consistency, with a Cronbach’s α coefficient of 0.91.

## Operational procedures

### Scale translation and cross-cultural adaptation

The researchers contacted the original author via email and obtained formal authorization from Professor Duygu Yildirim to use the English version of the scale. The translation and cross-cultural adaptation of the Scale of Perception and Understanding of Human Dignity in Nursing were conducted in accordance with the Brislin translation model [19]. Throughout the translation process, emphasis was placed on ensuring consistency of item meaning within the Chinese context, while preserving the original measurement intent and adapting the wording to the context of nursing education and practice in China. The adaptation process consisted of four stages: translation, back-translation, expert consultation, and pilot testing.

#### Translation and synthesis

Two nursing master’s students who had passed the College English Test Band 6 and possessed strong academic English reading skills independently translated the original English scale into Chinese, producing two initial versions (A1 and A2). Subsequently, the research team including nursing professors, a methodology expert, and the translators conducted item-by-item comparisons and discussions. Discrepancies were resolved through consensus, with careful consideration of professional nursing terminology and Chinese linguistic conventions, resulting in a synthesized Chinese draft (A1–2).

#### Blind back-translation

Two English-language researchers unfamiliar with the original scale and without medical backgrounds were invited to independently perform blind back-translation based solely on the Chinese draft, resulting in two back-translated versions (B1 and B2). The research team systematically compared the back-translated versions with the original English scale. For items where semantic deviations or expression differences were identified during back-translation such as the translation of core concepts like “autonomy” and “privacy” the team revisited the Chinese translations, refined the wording without departing from the original meaning, and reconfirmed the accuracy through an additional round of back-translation. The final revised Chinese version achieved high semantic consistency and natural expression.

#### Expert consultation and cultural adaptation

To ensure the content validity and contextual appropriateness of the scale within the Chinese cultural background, ten nursing experts with over ten years of clinical or teaching experience and intermediate or higher professional titles were invited to participate in the cultural adaptation assessment. The experts evaluated the items based on content relevance, cultural appropriateness, linguistic clarity, and clinical comprehensibility. During this process, the wording of certain items was optimized in response to expert recommendations to better align with clinical practice and ethical understanding in China. For example, Item 5, “Every individual is autonomous,” was initially translated literally as “Everyone is autonomous.” Experts pointed out that while autonomy in Western individualistic cultures emphasizes independent decision-making, in Chinas family-centered and communication-oriented healthcare culture, patient autonomy often manifests as shared decision-making based on informed consent. Therefore, this item was adapted to “Under the premise of informed consent and shared decision-making, every individual possesses autonomy,” making it more consistent with the provisions on patients right to informed consent in Chinas Civil Code and with clinical practice. Another significant adaptation involved Item 18, “Taking advantage of an individual’s weaknesses harms his dignity.” The literal translation, “Taking advantage of an individual’s weaknesses harms his dignity,” while ethically sound, was considered by multiple experts to be overly negative and direct in the context of traditional Chinese ethics emphasizing benevolence and self-discipline, as well as in the context of harmonious nurse-patient relationships. It also failed to clearly reflect the proactive protective responsibility of nursing staff. After several rounds of discussion with the expert panel and referencing the requirements of Chinas Regulations on Nursing such as respecting, caring for, and protecting patients, and safeguarding their privacy this item was revised to “In the nursing process, care should be taken to avoid compromising patient’s dignity due to their vulnerable circumstances.” This formulation aligns better with the implicit and positive approach to ethical instruction in Chinese culture and clarifies the proactive role of nursing staff in protecting patients dignity. The remaining items received high recognition from the experts in terms of semantic equivalence and cultural acceptability, with no fundamental modifications proposed.

#### Pilot testing

To evaluate the comprehensibility and acceptability of the scale, a convenience sampling method was used to recruit 20 nursing students for pilot testing. After completing the scale, participants took part in brief interviews to provide feedback on the clarity, ease of understanding, and potential semantic ambiguity of each item. The results indicated that all items were accurately understood, and the culturally adapted key items (such as Items 5 and 18) received positive feedback regarding linguistic naturalness and contextual relevance. Based on the interview feedback, minor adjustments were made to the wording of a few items to improve fluency and contextual fit, with all adjustments preserving the core meaning of the items.

For example, the initial translation of Item 41 was: “While providing nursing care effective communication should be established with individuals”. Some students noted that the term “individuals” felt somewhat abstract in the Chinese nursing context and was less specific than “patients”. Upon evaluation, the research team adopted this suggestion and refined the item to a more clinically conventional expression: “While providing nursing care, effective communication should be established with patients”. This modification only enhanced the specificity of the referent, while the core action of “establishing effective communication” and the measurement intent remained unchanged. Based on the pilot test results, the research team confirmed that the Chinese version of the scale is clearly expressed, semantically accurate, and suitable for subsequent formal investigation.

### Data collection

This study aimed to conduct a psychometric evaluation of the Chinese version of the Perception and Understanding of Human Dignity in Nursing Scale. For this purpose, a cross-sectional survey was employed as the data collection method. The electronic questionnaire was created and distributed via the Chinese online platform “Wenjuanxing.” Prior to the formal survey, the research team communicated with the heads of the nursing programs at the three universities. After obtaining their support, a convenience sampling method was used, with teachers from each university assisting in distributing the questionnaire link to nursing students. The first page of the questionnaire clearly stated the research purpose, the principle of voluntary participation, and data confidentiality measures. A total of 550 questionnaires were distributed. Invalid questionnaires were excluded based on the following criteria: [1] completion time less than 30 s; [2] answers displaying obvious response patterns (e.g., selecting the same option for multiple consecutive items). Ultimately, 530 valid questionnaires were retrieved, resulting in an effective response rate of 96.36%.

### Data analysis

This study employed statistical analysis software SPSS 25.0 and Amos 29.0 for data analysis. Count data were statistically described using frequency and composition ratio; continuous data meeting normal distribution were described using mean±standard deviation, while those not meeting normal distribution were described using median and interquartile range. Differences were considered statistically significant at *P* < 0.05. This study conducted item analysis, reliability analysis, and validity analysis on the Chinese version of the Scale Perception and Understanding of Human Dignity in Nursing.

### Item analysis

Cut-off Method: Rank scale scores from highest to lowest. Classify the top 27% of scorers as the high-score group and the bottom 27% as the low-score group. Compare the difference in scores between the two groups using an independent samples t-test. If the difference is not statistically significant (*P* > 0.05), it indicates poor discriminative power for that item, and deletion should be considered [20]. 2. Correlation Coefficient Method: Calculate the correlation coefficient between each item and the total scale score or corresponding subscales. If the correlation coefficient is ≤ 0.4 or is not statistically significant (*P* > 0.05), it indicates poor homogeneity between the item and the overall scale, warranting consideration for deletion [21]. 3. Cronbach’s α Coefficient Method: Calculate the overall Cronbach’s α coefficient for the scale. If α significantly increases after deleting an item, it indicates that the item negatively impacts the scale’s internal consistency and should be considered for deletion [22].

### Validity testing

1.Structural validity: The structural validity of the scale was assessed using Exploratory Factor Analysis (EFA) and Confirmatory Factor Analysis (CFA). In the structural exploration phase, exploratory factor analysis using principal component extraction was employed to examine the potential factor structure of the data. First, the Kaiser-Meyer-Olkin (KMO) test and Bartlett’s test of sphericity were conducted to evaluate the suitability of the data for factor analysis. Generally, a KMO value greater than 0.80 and a Bartlett’s test significance level of *P* < 0.001 indicate that the data are suitable for factor analysis [23]. In the factor analysis, factors were extracted based on eigenvalues greater than 1, and the results were interpreted in conjunction with the scree plot. The factor loading for each item was required to be no less than 0.50, and the cumulative variance explained was expected to be at least 50% in principle. Each factor was required to include no fewer than three items, with factor loadings no less than 0.40. For items with high loadings (> 0.40) on multiple factors and a difference of less than 0.20, whether to retain or remove them was determined based on theoretical relevance and statistical results [24]. It should be clarified that while principal component analysis is primarily a data reduction technique rather than a direct latent variable modeling approach, it was utilized in this study as an exploratory method to identify potential underlying structures within the scale items, with the understanding that subsequent confirmatory analysis would validate these structures. This analysis aimed to explore the potential factor structure of the data, providing a basis for subsequent model validation. On this basis, Confirmatory Factor Analysis (CFA) was further employed to test the structural model proposed based on the exploratory analysis. The goodness-of-fit indices for the model included χ²/df (CMIN/DF), the Root Mean Square Error of Approximation (RMSEA), the Standardized Root Mean Square Residual (SRMR), the Comparative Fit Index (CFI), the Normed Fit Index (NFI). Generally, a χ²/df < 3.00, RMSEA < 0.08, SRMR < 0.05, and CFI, NFI, IFI > 0.90 indicate an acceptable model fit [25]. 2. Content Validity: Content validity was assessed using the expert consultation method. Ten experts in the field of nursing were invited, including four nursing educators and six clinical nursing managers. All experts held at least a bachelor’s degree, had a professional title of associate senior or above, and had a minimum of 10 years of experience in nursing-related work. The researchers contacted the experts with the assistance of their supervisors and distributed and collected the consultation questionnaires via email. A total of 10 valid questionnaires were returned, with a 100% response and validity rate. Content validity was evaluated using the item-level content validity index (I-CVI) and the scale-level content validity index (S-CVI). The I-CVI represents the proportion of experts giving a rating of 3 or 4 for the same item, and the S-CVI is the average of all item I-CVI values. Generally, when the number of experts is six or more, an I-CVI ≥ 0.78 and S-CVI ≥ 0.90 indicate good content validity of the scale [26].

### Reliability testing

The reliability of the scale was evaluated through the following methods, (1) Internal Consistency: Cronbach’s α coefficient was calculated for the overall scale and for each of its dimensions. An α coefficient exceeding 0.700 indicates good internal consistency reliability [27]. (2) Split-Half Reliability: The scale items were divided into two halves (the first half and the second half) according to their sequential order in the questionnaire. The correlation coefficient between the scores of the two halves was then computed. A coefficient value greater than 0.700 is generally considered acceptable [28]. (3) Test-Retest Reliability: To examine the stability of the scale scores over time, a two-week follow-up survey was administered to the first 60 nursing students (IDs 1–60) who completed the initial questionnaire. This sample was selected because these participants were surveyed consecutively in the first wave, which facilitated tracking and contact. A two-week interval is commonly adopted in similar studies, as it helps reduce participants’ memory effect of the items while assuming that the measured psychological perception remains relatively stable over such a short period. Both surveys were conducted via the Wenjuanxing platform. Test-retest reliability was assessed by calculating the Pearson correlation coefficient between the total scores from the two test sessions. A correlation coefficient above 0.70, with a p-value less than 0.05, indicates acceptable temporal stability of the scale [29].

## Results

### Sample characteristics

A total of 550 questionnaires were distributed. After excluding invalid responses and participants who did not meet the inclusion criteria, 530 valid questionnaires were included for statistical analysis. The sample consisted predominantly of female nursing students (82.6%), with most participants aged 18–20 years (70.8%) and holding a bachelor’s degree (91.5%). Additionally, 70.0% of the nursing students reported having taken nursing ethics-related courses. Detailed demographic characteristics of the sample are presented in Table 1.

### Exploratory analysis: comparison of scores across different characteristics

To preliminarily understand the sample characteristics, exploratory analyses were conducted to examine differences in total scale scores and subscale scores across various demographic features. The results indicated statistically significant differences in the Perception dimension (F = 3.707, *p* = 0.025) and Care dimension (F = 5.789, *p* = 0.003) across different age groups. Similarly, statistically significant differences were observed in the total scale score (F = 6.983, *p* = 0.001) and across all three dimensions, Understanding (F = 8.329, *p* < 0.001), Perception (F = 4.622, *p* = 0.010), and Care (F = 5.331, *p* = 0.005) among nursing students of different educational levels (junior college, undergraduate, postgraduate). Specifically, undergraduate nursing students generally scored higher, whereas postgraduate students exhibited relatively lower total scale and subscale scores. It should be noted that these group differences are exploratory in nature, and their underlying reasons and clinical significance require further investigation. Detailed comparisons of scores across demographic characteristics, including means, standard deviations, and post hoc tests, are presented in Supplementary Material Table S1.

**Table 1:** Demographic characteristics frequency distribution (*n* = 530)

Demographic Variable	Category	Frequency (*n*)	Percentage (%)
Gender	Male	92	17.4
	Female	438	82.6
Age	18–20 years old	375	70.8
	21–23 years old	137	25.8
	≥ 24 years old	18	3.4
Education Level	Vocational College	35	6.6
	Undergraduate	485	91.5
	Graduate	10	1.9
Taken Nursing Ethics Course	Yes	371	70
	No	159	30

### Item analysis

#### Critical ratio method

The 530 participants were ranked based on their total scale scores from high to low. The top 27% (*n* = 143) were designated as the high-score group, and the bottom 27% (*n* = 143) as the low-score group. Independent samples t-tests were used to compare the differences in scores for each item between the two groups. The results showed that items A1, A2, A9, and A10 in the Understanding dimension had t-values below 3.00. Although the difference for item A2 was statistically significant (*p* < 0.05), its discrimination was relatively weak. For the remaining items, the score differences between the high- and low-score groups were statistically significant (|t| = 7.611–10.718, all *p* < 0.001), indicating good discrimination. Further examination of descriptive statistics revealed that these items with weaker discrimination exhibited marked negative skewness, with scores concentrated at the upper end of the scale (skewness ranging from − 0.439 to -0.544). The kurtosis values were close to or slightly above those of a normal distribution, suggesting the presence of a ceiling effect in responses to these items. This concentration of scores and negatively skewed distribution likely contributed to the limited differences between the high- and low-score groups, thereby affecting the discrimination performance of these items.

#### Item-total correlation analysis

Pearson correlation analysis was used to calculate the correlation coefficients between each item score and the total scale score. An item-total correlation coefficient of ≥ 0.30 is generally considered the minimum criterion for item retention. The results showed that the item-total correlation coefficients ranged from 0.325 to 0.738, all of which were statistically significant (*p* < 0.001), indicating good consistency between each item and the overall measurement objective of the scale. Even for items with lower discrimination in the critical ratio analysis, the item-total correlation coefficients remained within an acceptable range.

### Reliability analysis

Reliability analysis showed that the Cronbach’s α coefficient for the Understanding dimension was 0.916, with values ranging from 0.903 to 0.907 after deleting each item individually. The Cronbach’s α coefficient for the Perception dimension was 0.928, with values ranging from 0.918 to 0.925 after item deletion. For the Care dimension, the Cronbach’s α coefficient was 0.928, with values ranging from 0.920 to 0.925 after item deletion. These results indicate good internal consistency within each dimension, and deleting any item did not significantly improve the scale’s reliability. Based on the integrated results of critical ratio analysis, item-total correlation analysis, and reliability analysis, and considering the content significance and measurement objectives of the items, all 43 original items were retained in this study (see Table 2).

**Table 2:** Item analysis results of the Chinese version of the scale

Item	Mean	Standard Deviation	skewness	kurtosis	Critical Ratio C.*R*. (t)	*p*	Item-Total Correlation	Cronbach’s Alpha After Deletion
A1	4.360	0.607	-0.538	0.214	-2.620	0.009	0.423**	0.904
A2	4.360	0.599	-0.500	0.248	-1.716	0.087	0.350**	0.906
A3	4.840	0.445	-3.149	10.909	-6.933	0.000	0.610**	0.906
A4	4.840	0.409	-2.591	6.294	-7.611	0.000	0.646**	0.907
A5	4.830	0.439	-2.556	6.021	-8.381	0.000	0.668**	0.903
A6	4.830	0.408	-2.219	4.197	-8.772	0.000	0.659**	0.903
A7	4.810	0.457	-2.695	8.194	-8.057	0.000	0.562**	0.907
A8	4.830	0.400	-2.198	4.026	-9.468	0.000	0.619**	0.904
A9	4.330	0.600	-0.439	0.232	-2.435	0.016	0.424**	0.905
A10	4.340	0.612	-0.544	0.435	-2.588	0.010	0.397**	0.905
A11	4.820	0.419	-2.312	4.748	-9.856	0.000	0.690**	0.903
A12	4.840	0.420	-2.659	6.655	-8.871	0.000	0.684**	0.903
A13	4.790	0.468	-2.176	4.057	-10.065	0.000	0.619**	0.905
A14	4.820	0.463	-2.674	7.223	-10.593	0.000	0.652**	0.903
A15	4.780	0.468	-2.069	3.570	-10.718	0.000	0.617**	0.907
A16	4.770	0.520	-2.334	5.401	-10.274	0.000	0.586**	0.925
A17	4.810	0.440	-2.209	4.244	-10.182	0.000	0.657**	0.921
A18	4.800	0.455	-2.187	4.129	-10.481	0.000	0.647**	0.922
A19	4.820	0.437	-2.360	5.010	-8.658	0.000	0.631**	0.924
A20	4.820	0.439	-2.446	5.450	-9.867	0.000	0.684**	0.919
A21	4.840	0.422	-2.632	6.499	-8.152	0.000	0.627**	0.923
A22	4.820	0.445	-2.460	5.501	-8.508	0.000	0.620**	0.924
A23	4.820	0.450	-2.573	6.054	-8.961	0.000	0.635**	0.923
A24	4.820	0.461	-2.582	6.030	-9.398	0.000	0.682**	0.920
A25	4.820	0.457	-2.555	5.920	-9.766	0.000	0.724**	0.918
A26	4.770	0.493	-2.045	3.411	-11.090	0.000	0.653**	0.920
A27	4.800	0.448	-2.189	4.144	-9.408	0.000	0.642**	0.922
A28	4.800	0.475	-2.429	5.197	-9.642	0.000	0.618**	0.922
A29	4.780	0.495	-2.275	4.388	-10.022	0.000	0.625**	0.923
A30	4.810	0.454	-2.305	4.698	-8.307	0.000	0.583**	0.923
A31	4.840	0.422	-2.632	6.499	-8.890	0.000	0.617**	0.923
A32	4.790	0.458	-2.128	3.849	-9.792	0.000	0.636**	0.921
A33	4.820	0.453	-3.116	13.158	-8.142	0.000	0.564**	0.922
A34	4.840	0.430	-2.973	9.350	-8.560	0.000	0.646**	0.921
A35	4.830	0.493	-3.191	10.423	-7.403	0.000	0.649**	0.921
A36	4.830	0.452	-3.249	13.944	-8.361	0.000	0.687**	0.920
A37	4.790	0.502	-2.860	10.335	-8.932	0.000	0.561**	0.924
A38	4.770	0.482	-2.046	4.088	-9.576	0.000	0.601**	0.922
A39	4.830	0.446	-2.882	8.566	-6.971	0.000	0.558**	0.925
A40	4.810	0.494	-2.727	7.023	-8.644	0.000	0.638**	0.922
A41	4.860	0.399	-3.057	9.118	-8.500	0.000	0.738**	0.920
A42	4.840	0.439	-3.161	11.141	-8.228	0.000	0.659**	0.920
A43	4.810	0.457	-2.479	6.334	-8.969	0.000	0.638**	0.921

Note: All correlations were significant at *p* < 0.05. A1–A15 represent the 15 items of the understanding dimension, A16–A28 represent the 13 items of the perception dimension, and A29–A43 represent the 15 items of the care dimension

#### Content validity

This study assessed the authority of the experts using the expert authority coefficient (Cr). The results showed that the judgment basis coefficient (Ca) was 0.93, the judgment degree coefficient (Cs) was 0.83, and the comprehensive authority coefficient (Cr) was 0.88, all exceeding 0.8. Additionally, the Kendall’s W coefficient was 0.61, approaching 1, indicating relatively consistent and reliable consultation outcomes (see Table S2). The coefficient of variation ranged from 0 to 0.152, suggesting a high degree of agreement among the experts. The content validity of the Chinese version of the Perception and Understanding of Human Dignity in Nursing Scale was evaluated using a 1–4 rating scale (1 = completely irrelevant, 4 = completely relevant). The results showed that the item-level content validity index (I-CVI) was 1, and the scale-level average content validity index (S-CVI/Ave) was also 1, both exceeding 0.9, indicating good content validity (see Table S3). It should be noted that achieving I-CVI and S-CVI/Ave values of 1.0 is relatively uncommon in practical research, which primarily reflects a high level of agreement among experts in evaluating the items. However, this outcome may also be attributed to the fact that all participating experts were researchers in the field of nursing, resulting in a high degree of homogeneity in their core professional backgrounds. At the same time, the experts exhibited diversity in specific research areas (e.g., clinical nursing, nursing education, nursing management), years of clinical experience, and teaching backgrounds, which helps ensure the representativeness and reliability of the content validity assessment.

#### Construct validity

##### Exploratory factor analysis

**Fig. 1:**
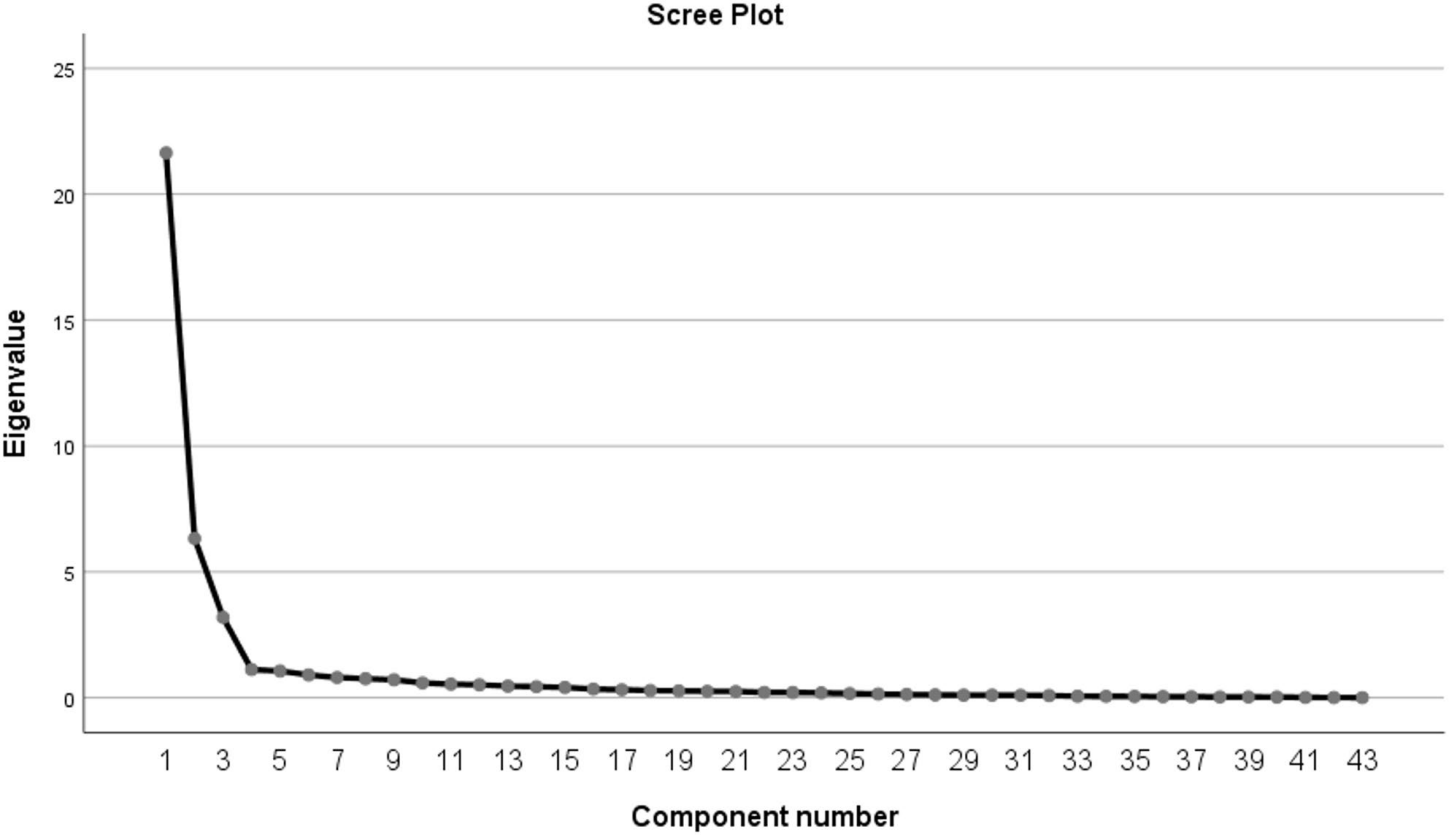
Scree plot

Exploratory factor analysis (EFA) was conducted using SPSS 25.0 on the first randomly selected half of the sample (*n* = 265). The Kaiser–Meyer–Olkin (KMO) measure of sampling adequacy was 0.919, and Bartlett’s test of sphericity yielded χ² = 17,698.66 (*p* < 0.001), indicating that the data were suitable for factor analysis (see Table S4). Principal component extraction-based EFA was performed, followed by varimax orthogonal rotation. Three factors were extracted, corresponding to the original scale’s dimensions of “Understanding,” “Perception,” and “Care.” The assignment of items to factors was consistent with the original scale. The factor loadings of the items on their corresponding factors ranged from 0.518 to 0.947, all exceeding the retention threshold of 0.50 and meeting statistical standards (see Table 3). The overall high factor loadings (with many items exceeding 0.90) suggest good construct consistency of the scale but may also indicate some degree of measurement content overlap among certain items. Items 16 (0.548) and 19 (0.518) had relatively lower loadings but were retained due to their clear theoretical relevance and alignment with the original scale’s factor structure. Cross-loadings of all items were examined, and no item exhibited cross-loadings exceeding 0.40 on multiple factors, indicating that the items primarily loaded onto their respective dimensions. The scree plot of the scale is shown in Fig. 1.

**Table 3:** Rotated factor loadings of exploratory factor analysis (*N* = 265)

Item	Statement	F1	F2	F3
1	Every individual is free.	0.899		
2	Every individual is special.	0.885		
3	Every individual is equal.	0.630		
4	Every individual is valuable.	0.644		
5	Under the premise of informed consent and shared decision-making, every individual possesses autonomy	0.944		
6	Every individual should lead a life within the framework of the principle of social justice.	0.925		
7	Every individual has certain values.	0.573		
8	Every individual has the right to physical and moral inviolability.	0.947		
9	Every individual has the right to honor and dignity.	0.932		
10	Every individual has the right to live in an environment that complies with the principle of justice brought by law.	0.914		
11	Every individual wants to protect his dignity and not give up on it.	0.883		
12	Human dignity is inviolable.	0.906		
13	The individuality/singularity of each individual must be respected.	0.851		
14	The identity of each individual must be respected.	0.699		
15	The autonomy of each individual must be respected.	0.905		
16	Every individual has moral value judgments that he or she adopts.			0.548
17	Exposure of an individual to discriminatory behavior damages his dignity as a human being.			0.872
18	In the nursing process, care should be taken to avoid compromising patients’ dignity due to their vulnerable circumstances			0.880
19	The dignity of each individual is inviolable.			0.518
20	A person’s self-respect is possible by feeling that he is an honorable being.			0.912
21	Each individual is responsible for protecting his own dignity.			0.562
22	Society has a responsibility to protect the dignity of individuals.			0.616
23	The protection of the right to life is a necessity to protect human dignity.			0.563
24	It is necessary to respect each individual’s own self-worth.			0.858
25	Every individual’s exposure to any form of physical violence negatively affects the perception of dignity.			0.910
26	Every individual’s exposure to any form of psychological violence negatively affects the perception of honor.			0.824
27	An individual’s perception of dignity may be affected by ethical and moral values.			0.803
28	Providing information support to each individual during the treatment and care process positively affects their perception of dignity.			0.832
29	The privacy of each individual must be respected.		0.656	
30	Every individual has the right to choose their health care practices.		0.643	
31	Every individual should have the right to decide for medical interventions on his body.		0.647	
32	Every patient has the right to good nursing care.		0.726	
33	Every individual has the right to die with dignity under good nursing care.		0.822	
34	Respecting the dignity of the individual’s existence is the foundation of nursing care.		0.800	
35	It is important to protect the secrets and privacy of each individual.		0.862	
36	Protecting the dignity of each individual is the ethical responsibility of nurses.		0.733	
37	The preferences of the individual should be taken into account when providing nursing care.		0.660	
38	The principle of fair treatment is essential in nursing care.		0.736	
39	Care should be taken to protect the privacy of the individual while giving nursing care.		0.692	
40	Confidentiality of the individual should be taken into consideration while giving nursing care.		0.730	
41	While providing nursing care, effective communication should be established with patients.		0.690	
42	The unchanging principle of nursing care is the protection of human dignity.		0.802	
43	It is human dignity to give appropriate nursing care to each individual throughout his illness.		0.713	

Note: Extraction method: Principal Component Analysis (PCA). Rotation method: Kaiser normalized maximum variance method. A rotation converged after 5 iterations. F1 represents the Understanding Dimension, F2 represents the Perception Dimension, and F3 represents the Nursing Dimension

### Confirmatory factor analysis

**Fig. 2:**
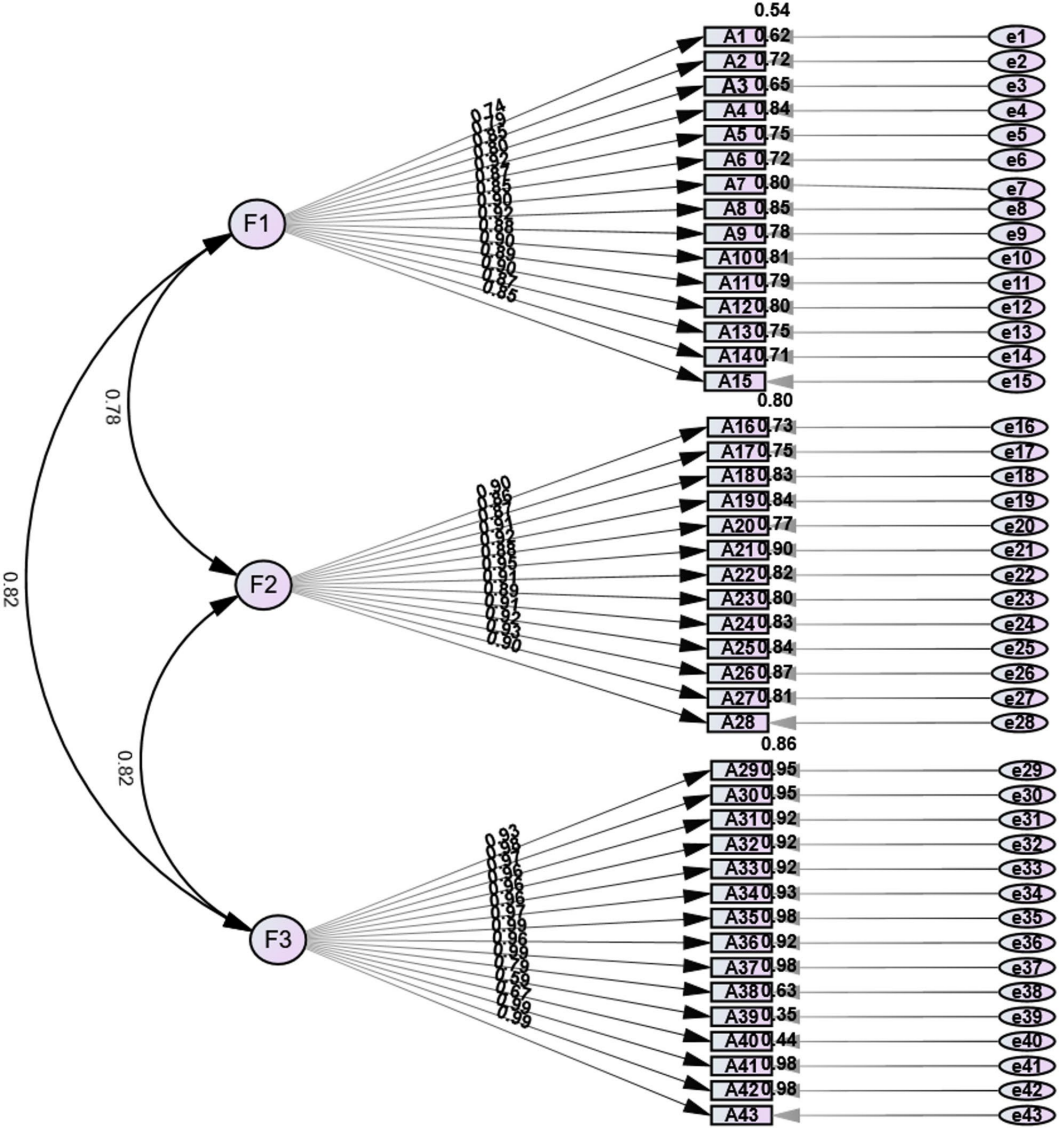
Standardized three-factor structural equation model (*n* = 265)

To examine the structural validity of the scale, confirmatory factor analysis (CFA) was conducted using the other independent random half of the sample (*n* = 265), with maximum likelihood as the parameter estimation method. Based on the EFA results, a three-factor structural model for the Chinese version of the Perception and Understanding of Human Dignity in Nursing Scale was constructed (see Fig. 2). Confirmatory factor analysis demonstrated an acceptable overall model fit, with all key indices meeting standard thresholds (see Table 4). Notably, the normed fit index (NFI) was slightly below 0.90 but remained within an acceptable range. The standardized factor loadings of all items on their respective latent variables were statistically significant (*p* < 0.001), including acceptable loadings for items with lower discrimination in item analysis (e.g., A2), indicating that the items adequately reflected the corresponding latent constructs. The composite reliability (CR) values for each factor ranged from 0.977 to 0.988, all exceeding the recommended threshold of 0.70. The average variance extracted (AVE) values ranged from 0.744 to 0.816, all surpassing the recommended threshold of 0.50. The high CR and AVE values indicate excellent internal consistency and convergent validity, demonstrating that the items effectively reflect their corresponding constructs. However, they also suggest the possibility of some degree of measurement content overlap or redundancy among scale items, potentially limiting the breadth of construct measurement. Furthermore, the square roots of the AVE values for each construct were greater than their correlations with other constructs (see Tables 5 and 6), indicating good convergent and discriminant validity of the scale.

**Table 4:** Model fit indices

Adaptation Metrics	χ² / df	RMSEA	SRMR	CFI	NFI
Measurement Value	2.566	0.077	0.014	0.933	0.894
Scope	<3.000	<0.080	<0.050	>0.900	>0.800

Note: χ²/df = Chi-square/degrees of freedom ratio, RMSEA = Root Mean Square

Error of Approximation, SRMR = Standardized Root Mean Square Residual,

CFI = Comparative Fit Index, NFI = Normative Fit Index

**Table 5:** Convergent validity

			Standardized factor loadings	S.E.	*P*	CR	AVE
A1	<---	F1	0.737			0.977	0.744
A2	<---	F1	0.790	0.085	***		
A3	<---	F1	0.848	0.076	***		
A4	<---	F1	0.805	0.079	***		
A5	<---	F1	0.918	0.070	***		
A6	<---	F1	0.868	0.071	***		
A7	<---	F1	0.847	0.071	***		
A8	<---	F1	0.896	0.068	***		
A9	<---	F1	0.921	0.072	***		
A10	<---	F1	0.885	0.069	***		
A11	<---	F1	0.899	0.073	***		
A12	<---	F1	0.888	0.077	***		
A13	<---	F1	0.897	0.068	***		
A14	<---	F1	0.868	0.073	***		
A15	<---	F1	0.846	0.069	***		
B1	<---	F2	0.895			0.983	0.816
B2	<---	F2	0.856	0.038	***		
B3	<---	F2	0.865	0.046	***		
B4	<---	F2	0.913	0.035	***		
B5	<---	F2	0.919	0.034	***		
B6	<---	F2	0.879	0.040	***		
B7	<---	F2	0.950	0.030	***		
B8	<---	F2	0.906	0.041	***		
B9	<---	F2	0.894	0.043	***		
B10	<---	F2	0.914	0.040	***		
B11	<---	F2	0.916	0.035	***		
B12	<---	F2	0.935	0.034	***		
B13	<---	F2	0.899	0.035	***		
C1	<---	F3	0.925			0.988	0.847
C2	<---	F3	0.975	0.030	***		
C3	<---	F3	0.973	0.030	***		
C4	<---	F3	0.960	0.032	***		
C5	<---	F3	0.959	0.032	***		
C6	<---	F3	0.959	0.032	***		
C7	<---	F3	0.967	0.031	***		
C8	<---	F3	0.990	0.027	***		
C9	<---	F3	0.958	0.032	***		
C10	<---	F3	0.990	0.027	***		
C11	<---	F3	0.794	0.054	***		
C12	<---	F3	0.590	0.081	***		
C13	<---	F3	0.667	0.070	***		
C14	<---	F3	0.990	0.027	***		
C15	<---	F3	0.990	0.027	***		

Note: *** indicates *P* ≤ 0.01, with statistically significant differences

F1 represents the dimension of understanding, F2 represents the dimension of care, F3 represents the dimension of perception, A1-A15 represent 15 items of the dimension of understanding, B1-B13 represent 13 items of the dimension of perception, and C1-C15 represent 15 items of care

**Table 6:** Distinctive validity

F1	F1	F2	F3
	0.863		
F2	0.783*	0.903	
F3	0.821*	0.823**	0.920
AVE	0.744	0.816	0.847

Note: * indicates < 0.05, and ** indicates < 0.01

### Reliability analysis

The reliability of the scale was evaluated across three domains: internal consistency, split-half reliability, and test-retest reliability. The scale demonstrated excellent internal consistency, with a Cronbach’s α of 0.958 for the total score and subscale values ranging from 0.916 to 0.928 (Understanding, Perception, Care), all exceeding the 0.90 benchmark for excellent reliability (Table 7). Split-half reliability was also strong. Both the Spearman-Brown corrected coefficient and the Guttman split-half coefficient were 0.836, surpassing the acceptable threshold of 0.80 (see Table S5). Test-retest reliability over a two-week interval was assessed using Pearson’s correlation coefficient (r) and the intraclass correlation coefficient (ICC) to evaluate temporal stability from both correlation and agreement perspectives. Results indicated exceptionally high short-term stability for the total scale, Pearson *r* = 0.966 and ICC = 0.971. Further analysis revealed that the average-measure ICC values for all subscales and the total scale were no lower than 0.943, with the lower bounds of the 95% confidence intervals all above 0.854. All corresponding F-tests were statistically significant (*p* < 0.001), providing robust evidence for the scale’s short-term stability (Table S6). It should be noted that while these high coefficients reflect strong short-term reliability, the potential influence of memory effects cannot be entirely ruled out given the two-week retest interval. Future studies could consider extending the retest interval to more comprehensively examine the scale’s temporal reliability and control for potential memory effects.

**Table 7:** Reliability analysis

Scale Dimensions	Cronbach’s coefficient	Split-half reliability	Reliability of retesting
Understanding	0.916		0.978
Perception	0.928		0.972
Care	0.928		0.919
Total	0.958	0.836	0.966

## Discussion

Cultivating nursing students’ perception and understanding of human dignity is one of the core objectives of nursing ethics education. Dignity-centered nursing practice, which emphasizes respect for patient privacy, autonomy, and fair treatment, has been demonstrated to correlate with improved patient experiences and satisfaction [30, 31]. However, in real-world clinical environments, various factors may hinder the full implementation of these ideals [32]. Therefore, developing and validating a measurement tool suitable for China’s nursing education context holds practical significance for systematically assessing nursing students’ cognitive levels of human dignity and providing evidence-based guidance for ethics education.

From a psychometric perspective, item analysis demonstrated that most items exhibited good discriminative power and homogeneity. Notably, some item scores displayed a negatively skewed distribution and clustered toward the higher end, suggesting a potential ceiling effect. This phenomenon may reflect respondents’ high normative endorsement of the ethical ideal of “human dignity” rather than directly equating to their actual behavioral performance in clinical contexts. It may also be associated with social desirability bias. Validity analysis supported the three-factor structure of the scale, with both exploratory and confirmatory factor analysis results aligning with the theoretical framework. However, certain indicators warrant deeper interpretation. The overall high factor loadings, along with the exceptionally high composite reliability (CR) and average variance extracted (AVE) values, confirm the strong internal consistency among the scale items. On the other hand, they also suggest potential content overlap or measurement redundancy among the items, indicating that the breadth of the measured construct may be relatively narrow. In terms of reliability, the scale demonstrated good internal consistency and short-term test-retest stability.

At a practical level, this Chinese version of the scale can serve as an auxiliary tool to monitor the development of nursing students’ ethical cognition and provide references for curriculum design and instructional improvements. It should be noted that 70.8% of participants in this study were younger students aged 18–20 years. This age distribution may influence the interpretation of the findings. Compared to senior students or clinical nurses, these younger students have relatively limited clinical exposure and experience. Their perception of human dignity may therefore be based more on theoretical understanding and idealized attitudes formed through classroom learning, rather than on practical reflection gained from handling complex clinical ethical dilemmas. Consequently, the developmental stage of students’ cognition should be considered in educational applications. For instance, in early undergraduate education, scale assessments could be effectively combined with scenario simulations and reflective discussions to foster students’ value recognition of the concept of dignity. For students in clinical practicum, graduate, or continuing education stages, the educational focus could shift towards guiding them to analyze the application of and challenges to dignity principles in specific, complex clinical contexts (e.g., end-of-life decision-making, resource allocation), thereby facilitating the transformation of ethical cognition into clinical decision-making capability.

From a cross-cultural perspective, the findings of this study support the measurability of the construct “nursing students’ perception and understanding of human dignity” across cultural contexts. The core values measured by the scale dignity, autonomy, and fairness possess universal ethical significance. However, their specific connotations, relative importance, and practical expressions are profoundly influenced by cultural contexts. China’s collectivist cultural values emphasize harmony, relationships, and social roles, which may lead individuals to perceive human dignity more in terms of respect gained within interpersonal relationships and social evaluation networks. This differs from the Western cultural framework of dignity, which centers on individual autonomy and independence. The cultural adaptation work of this scale aims to capture this potential cultural specificity.

Future research could employ methods such as Item Response Theory on broader samples to conduct more refined testing and optimization of the items’ measurement performance. The long-term stability of the scale over time and its sensitivity to educational interventions require further validation through extended test-retest intervals or longitudinal designs. Its cross-cultural measurement equivalence also still requires rigorous examination through direct comparisons of multicultural samples.

### Limitations

The interpretation and generalizability of this study’s findings should be considered in light of the following limitations. First, the sample’s representativeness is constrained. Participants were recruited from three universities in Shandong and Liaoning provinces using convenience sampling. Although the sample covered various educational levels (including junior college, undergraduate, and postgraduate) and spanned two geographical regions, the sampling approach limits its representativeness of the broader population of nursing students in China. It should be emphasized that this study did not test measurement invariance across different educational subgroups (junior college, undergraduate, postgraduate). Therefore, comparisons of score differences among these groups should be made with caution. Additionally, the sample was relatively young (70.8% aged 18–20 years). Their limited clinical experience may influence the depth of their perception regarding dignity issues in nursing practice, and caution is advised when generalizing the findings to older or more experienced nursing student groups.

Second, limitations exist regarding the study population and methodology. In line with the original scale’s intended target, this study exclusively included nursing students. Consequently, the findings cannot be directly extrapolated to practicing clinical nurses, and the scale’s applicability in clinical work environments requires further validation. All data were collected via self-report questionnaires, which may introduce common method bias. Self-report scales measuring ethical cognition, in particular, may be more susceptible to social desirability bias. Future research could consider integrating multiple data sources, such as behavioral observations and scenario-based assessments, to enhance the validity of the findings.

Third, there are psychometric limitations. In the exploratory factor analysis, principal component analysis (PCA) was employed for factor extraction. While this is a common practice, it differs theoretically from extraction methods based on common factor models. More notably, this study observed exceptionally high internal consistency reliability and short-term test-retest reliability. Although these indicate good measurement precision, the high coefficients may stem from item content redundancy. Furthermore, the short retest interval (two weeks) cannot completely rule out the potential influence of memory effects, representing another methodological limitation of this study. The scale’s long-term stability requires further verification with more diverse samples. Finally, this study has not yet examined the criterion-related validity of the scale. Its effectiveness in predicting nursing students’ actual ethical behavior or clinical humanistic care competence remains unknown. Future research should establish this by correlating the scale with established behavioral observation tools or performance evaluations.

Future studies should expand sampling to include a wider range of geographical areas and types of institutions and extend validation to clinical nurse populations. Employing multi-method data collection strategies can help reduce common method bias. On the basis of testing measurement invariance, in-depth comparisons across different groups and over time should be conducted. Ultimately, through the examination of criterion validity, the practical application value of this scale in nursing education quality assessment and personnel selection can be established.

## Conclusion

Given the lack of a specific measurement tool for assessing nursing students’ perception and understanding of human dignity in the Chinese nursing context, this study introduced and systematically adapted the Turkish-developed Scale of Perception and Understanding of Human Dignity in Nursing, followed by psychometric validation. The results indicate that the Chinese version of the scale demonstrates acceptable reliability and validity metrics among nursing students in China. In summary, this study provides preliminary empirical support for the use of this scale within the Chinese nursing student population. These findings offer an initial reference for the exploratory application of this scale in the Chinese nursing education context to assess students’ perception and understanding of dignity. Future research should further verify its measurement properties in broader and more diverse samples and explore its specific application value in educational assessment and practical intervention.

## Supplementary Information

Below is the link to the electronic supplementary material.

Supplementary Material 1

## Data Availability

The dataset used and/or analyzed during the current study period can be obtained from the corresponding author upon reasonable request.

## Abbreviations

AVE: Average Variance Extracted
CFA: Confirmatory Factor Analysis
CFI: Comparative Fit Index
CR: Composite Reliability
Cronbach‘s α: Cronbach‘s Alpha Coefficient
CVI: Content Validity Index
EFA: Exploratory Factor Analysis
I-CVI: Item-level Content Validity Index
IFI: Incremental Fit Index
KMO: Kaiser-Meyer-Olkin (Measure of Sampling Adequacy)
NFI: Normed Fit Index
PCA: Principal Component Analysis
RMSEA: Root Mean Square Error of Approximation
RMR: Root Mean Square Residual
