# Supplementary material for: Psychometric evaluation and cross-cultural adaptation of the Chinese version of the Perception and Understanding of Human Dignity in Nursing Scale: a methodological study
Source: BMC Nurs. 2026 Mar 7;25:360. doi: 10.1186/s12912-026-04525-y (PMC13081378; doi:10.1186/s12912-026-04525-y)
Supplement: Supplementary file 1 — Supplementary Material 1 [file 12912_2026_4525_MOESM1_ESM.docx]

| **Table S1.** Scores on the Scale of Perception and Understanding of Human Dignity among nursing students with different characteristics (n=530) | | | | | | |  |
| --- | --- | --- | --- | --- | --- | --- | --- |
|  |  |  | Mean ± SD / Statistic (p-value) | | | | |
| Characteristic Variable | Group | n | Total Scale | Understanding Dimension | Perception Dimension | Nursing Dimension | |
| Gender | Male | 92 | 203.910±14.552/-0.956（0.341） | 70.033±5.638/-0.743（0.459） | 62.435±5.056/-0.158（0.874） | 71.446±5.754/-1.560(0.121) | |
|  | Female | 438 | 205.460±11.600 | 70.500±4.693 | 62.514±4.193 | 72.443±4.615 | |
| Nursing Ethics Course | Yes | 371 | 205.150±12.142/-0.109(0.913) | 70.388±4.851/-0.222(0.825) | 62.539±4.429/0.316(0.752) | 72.2237±4.960/-0.335(0.738) | |
|  | No | 159 | 205.280±12.249 | 70.491±4.919 | 62.409±4.171 | 72.377±4.564 | |
| Age | 18-20 | 375 | 205.970±11.1280/2.667(0.070) | 70.485±4.678/0.486(0.615) | 62.813±3.978/3.707(0.025) | 72.672±4.398/5.789(0.003) | |
|  | 21-23 | 137 | 203.300±14.230 | 70.380±5.425 | 61.847±4.808 | 71.073±5.717 | |
|  | ≥24 | 18 | 203.280±14.596 | 69.333±4.352 | 60.944±6.898 | 73.000±5.145 | |
| Educational Background | Junior College | 35 | 203.970±20.763/6.983(0.001) | 68.6±7.523/8.329(0.000) | 62.4±6.976/4.622(0.010) | 72.9714±6.684/5.331(0.005) | |
|  | Bachelor's Degree | 485 | 205.56±10.988 | 70.6515±4.509 | 62.5918±3.976 | 72.3175±4.574 | |
|  | Postgraduate | 10 | 191.4±18.698 | 65.5±6.553 | 58.4±7.677 | 67.5±7.502 | |

**Table S2.** Results of Expert Consultation

| Expert | Theoretical Knowledge | Practical Experience | Reference to Domestic and International Materials | Personal Intuition | Familiarity Level | Ca | Cs | Cr | Kendall's Coefficient of Agreement |
| --- | --- | --- | --- | --- | --- | --- | --- | --- | --- |
| 1 | 0.3 | 0.5 | 0.1 | 0.1 | 1.0 | 0.93 | 0.83 | 0.88 | 0.61 |
| 2 | 0.2 | 0.5 | 0.1 | 0.1 | 0.5 |  |  |  |  |
| 3 | 0.3 | 0.3 | 0.1 | 0.1 | 0.2 |  |  |  |  |
| 4 | 0.3 | 0.5 | 0.1 | 0.1 | 0.8 |  |  |  |  |
| 5 | 0.1 | 0.5 | 0.1 | 0.1 | 1.0 |  |  |  |  |
| 6 | 0.3 | 0.5 | 0.1 | 0.1 | 1.0 |  |  |  |  |
| 7 | 0.3 | 0.5 | 0.1 | 0.1 | 0.8 |  |  |  |  |
| 8 | 0.3 | 0.5 | 0.1 | 0.1 | 1.0 |  |  |  |  |
| 9 | 0.2 | 0.4 | 0.1 | 0.1 | 1.0 |  |  |  |  |
| 10 | 0.3 | 0.5 | 0.1 | 0.1 | 1.0 |  |  |  |  |

| **Table S3.** Content Validity | | | | | | |
| --- | --- | --- | --- | --- | --- | --- |
| Item | Standard Deviation | Minimum | Maximum | Coefficient of Variation | I-CVI | S-CVI/Ave |
| A1 | 0.000 | 4.000 | 4.000 | 0.400 | 1.000 | 1.000 |
| A2 | 0.000 | 4.000 | 4.000 | 0.400 |  |  |
| A3 | 0.000 | 4.000 | 4.000 | 0.400 |  |  |
| A4 | 0.000 | 4.000 | 4.000 | 0.400 |  |  |
| A5 | 0.000 | 4.000 | 4.000 | 0.400 |  |  |
| A6 | 0.000 | 4.000 | 4.000 | 0.400 |  |  |
| A7 | 0.527 | 3.000 | 4.000 | 0.350 |  |  |
| A8 | 0.000 | 4.000 | 4.000 | 0.400 |  |  |
| A9 | 0.000 | 4.000 | 4.000 | 0.400 |  |  |
| A10 | 0.000 | 4.000 | 4.000 | 0.400 |  |  |
| A11 | 0.000 | 4.000 | 4.000 | 0.400 |  |  |
| A12 | 0.000 | 4.000 | 4.000 | 0.400 |  |  |
| A13 | 0.422 | 3.000 | 4.000 | 0.320 |  |  |
| A14 | 0.000 | 4.000 | 4.000 | 0.400 |  |  |
| A15 | 0.000 | 4.000 | 4.000 | 0.400 |  |  |
| A16 | 0.000 | 4.000 | 4.000 | 0.400 |  |  |
| A17 | 0.000 | 4.000 | 4.000 | 0.400 |  |  |
| A18 | 0.516 | 3.000 | 4.000 | 0.360 |  |  |
| A19 | 0.000 | 4.000 | 4.000 | 0.400 |  |  |
| A20 | 0.483 | 3.000 | 4.000 | 0.330 |  |  |
| A21 | 0.000 | 4.000 | 4.000 | 0.400 |  |  |
| A22 | 0.000 | 4.000 | 4.000 | 0.400 |  |  |

**Continued TableS3**. Content Validity

| Item | | Standard Deviation | | | Minimum | Maximum | | Coefficient of Variation | | | I-CVI | | S-CVI/Ave |
| --- | --- | --- | --- | --- | --- | --- | --- | --- | --- | --- | --- | --- | --- |
| A23 | | 0.000 | 4.000 | | | 4.000 | | 0.400 |  | |  | |  |
| A24 | | 0.000 | 4.000 | | | 4.000 | | 0.400 |  |  |  |  |  |
| A25 | | 0.000 | 4.000 | | | 4.000 | | 0.400 |  |  |  |  |  |
| A26 | | 0.000 | 4.000 | | | 4.000 | | 0.400 |  |  |  |  |  |
| A27 | | 0.000 | 4.000 | | | 4.000 | | 0.400 |  |  |  |  |  |
| A28 | | 0.000 | 4.000 | | | 4.000 | | 0.400 |  |  |  |  |  |
| A29 | | 0.000 | 4.000 | | | 4.000 | | 0.400 |  |  |  |  |  |
| A30 | | 0.000 | 4.000 | | | 4.000 | | 0.400 |  |  |  |  |  |
| A31 | | 0.000 | 4.000 | | | 4.000 | | 0.400 |  |  |  |  |  |
| A32 | | 0.000 | 4.000 | | | 4.000 | | 0.400 |  |  |  |  |  |
| A33 | | 0.000 | 4.000 | | | 4.000 | | 0.400 |  |  |  |  |  |
| A34 | | 0.000 | 4.000 | | | 4.000 | | 0.400 |  |  |  |  |  |
| A35 | | 0.000 | 4.000 | | | 4.000 | | 0.400 |  |  |  |  |  |
| A36 | | 0.000 | 4.000 | | | 4.000 | | 0.400 |  |  |  |  |  |
| A37 | | 0.000 | 4.000 | | | 4.000 | | 0.400 |  |  |  |  |  |
| A38 | | 0.000 | 4.000 | | | 4.000 | | 0.400 |  |  |  |  |  |
| A39 | | 0.000 | 4.000 | | | 4.000 | | 0.400 |  |  |  |  |  |
| A40 | | 0.000 | 4.000 | | | 4.000 | | 0.400 |  |  |  |  |  |
| A41 | | 0.000 | 4.000 | | | 4.000 | | 0.400 |  |  |  |  |  |
| A42 | | 0.000 | 4.000 | | | 4.000 | | 0.400 |  |  |  |  |  |
| A43 | | 0.516 | 3.000 | | | 4.000 | | 0.340 |  |  |  |  |  |

| **Table S4.** KMO and Bartlett's Test | | |
| --- | --- | --- |
| KMO Sampling Adequacy Measure |  | 0.919 |
| Bartlett's Sphericity Test | Approximate chi-square | 17698.66 |
|  | degree of freedom | 903 |
|  | P | 0.000 |

**Table S****5.** Split-Half Reliability

| Klonbach Alpha | | | | Spearman-Brown coefficient | |
| --- | --- | --- | --- | --- | --- |
| Part I (22 Questions) | | Part II (21 Questions) | |  |  |
| 0.924 |  | 0.943 |  | 0.836 |  |

| **Table S6.** Intraclass Correlation Coefficient | | | | | | | | |
| --- | --- | --- | --- | --- | --- | --- | --- | --- |
|  |  |  | 95% CI | | F-test with a true value of 0 | |  |  |
| Dimension |  | Intraclass Correlationᵇ | Lower Bound | Upper Bound | Value | df1 | ddf2 | Significance |
| Understanding | Average Measures | 0.983^c^ | 0.942 | 0.986 | 88.053 | 59 | 59 | 0 |
| Perception | Average Measures | 0.983^c^ | 0.964 | 0.991 | 69.287 | 59 | 59 | . |
| Care | Average Measures | 0.943^c^ | 0.854 | 0.973 | 23.227 | 59 | 59 | 0 |
| Total Scale | Average Measures | 0.971^c^ | 0.854 | 0.989 | 58.121 | 59 | 59 | 0 |
| A two-way mixed-effects model where the person effect is random and the measurement effect is fixed. a. The estimator remains the same regardless of the presence of interaction effects. b. Intraclass correlation coefficient (ICC) of Class A defined using absolute agreement. c. This estimation is calculated under the assumption of no interaction effects; otherwise, it cannot be estimated. | | | | | | | | |
